# Meal Pattern Variables and 15-Year Mortality: Results from the Gothenburg H70 Birth Cohort Studies, Sweden

**DOI:** 10.29219/fnr.v69.11445

**Published:** 2025-05-08

**Authors:** Emmalee Gisslevik, Love Svanqvist, Ingmar Skoog, Lauren Lissner, Elisabet Rothenberg

**Affiliations:** 1 Faculty of Health Sciences, Kristianstad University, Kristianstad, Sweden; 2 Neuropsychiatric Epidemiology Unit, Department of Psychiatry and Neurochemistry, Institute of Neuroscience and Physiology, Sahlgrenska Academy, Centre for Ageing and Health (AgeCap) at the University of Gothenburg, Gothenburg, Sweden; 3 Department of Community Medicine and Public Health, at the University of Gothenburg, Gothenburg, Sweden

**Keywords:** meal patterns, all-cause mortality, eating frequency, dietary habits, geriatric health, ageing population, survival analysis, longitudinal study, Cox regression

## Abstract

**Background:**

Dietary habits throughout life significantly influence health in old age; yet, little is known about the relationship between meal pattern and mortality among older adults.

**Objective:**

This study aimed to prospectively investigate the association between meal pattern variables and 15-year all-cause mortality in a cohort of 70-year-olds from the Gothenburg H70 study, considering relevant covariates.

**Design:**

A total of 551 individuals (321 women and 230 men) were included. Dietary intake was assessed using the diet history method, reflecting intake over the preceding 3 months. Meal patterns were described by the usual daily frequency of main meals, light meals, snacks, beverages, and total intake occasions (IO). Statistical analyses included Cox proportional hazards regression, Student’s *t*-test, and Chi-square test.

**Results:**

Subjects who were deceased at follow-up had a higher prevalence of undernutrition risk indicators (based on low body mass index [BMI], weight/appetite change, and eating difficulties) at baseline compared to those living 15 years later (*P* = 0.02). In the fully adjusted Cox model, individuals with high total intake frequencies (>5 per day) showed a significantly increased hazard ratio (1.51) for mortality compared to those with medium frequencies. Additionally, medium-high snack frequency (>2–3 snacks/day) was associated with an elevated mortality risk, independent of total energy intake and other covariates.

**Discussion:**

These findings suggest a potential association between frequent daily IO, particularly snacks, and increased mortality risk, which is not fully explained by total energy consumption or other covariates.

**Conclusions:**

The 15-year follow-up provides a long-term view of meal patterns’ impact on longevity, indicating that higher daily consumption frequencies may be associated with increased mortality risk between ages 70 and 85. Further research should examine the nutritional composition of various meal patterns to clarify these associations.

## Popular scientific summary

- This study explores the link between meal frequency and long-term mortality risk in older adults.
- Findings suggest that higher meal frequencies may be associated with increased mortality, independent of total energy intake.
- Results highlight the need for further research on meal patterns, considering both frequency and nutritional quality.
- Insights from this study could inform dietary guidelines, especially for ageing populations at risk of malnutrition and metabolic disorders.

Life expectancy has increased in Europe as well as globally, with older adults living longer due to lifestyle changes, improved living conditions, and better health (1–4). In the Gothenburg H70 Birth Cohort Studies, positive health trends have been demonstrated in five birth cohorts examined at the same age over the past 50 years, with later-born cohorts generally at better health (5–9). As one example, today’s 70 year-olds show healthier dietary patterns compared to earlier cohorts (10–12).

However, the older population is characterized by significant heterogeneity in terms of health and vigour. Health trajectories evolve differently between individuals, being influenced by factors such as lifestyle, socioeconomic factors, and genetics (13). Non-communicable diseases (NCDs) – chronic conditions such as cardiovascular diseases, cancers, and diabetes – are prevalent among older adults and are often associated with lifestyle factors such as suboptimal diet and physical inactivity (14).

Suboptimal diets increase the risk of malnutrition, encompassing an associated shift from overnutrition (i.e. a risk of overweight and obesity) to undernutrition (risk of insufficient energy/nutrients) with advancing age (15, 16). In later life, undernutrition becomes particularly concerning due to its associations with higher infection risk, prolonged recovery, increased mortality, and reduced quality of life. Even though multiple physiological, social, and behavioural factors contribute to these challenges, diet remains a common denominator that can either alleviate or exacerbate malnutrition in all its forms, underscoring the importance of addressing dietary patterns for healthy ageing (16).

Traditionally, research on diet-disease relationships has focused on specific nutrients and their imbalances, overlooking the interactive nature of nutrients within whole foods. Additionally, people consume a wide range of food items, making it crucial to examine the role of overall diet and food patterns in preventing diet-related morbidity and mortality (17), an area that has been the focus of recent nutrition research (18, 19).

Moreover, dietary intake is commonly grouped into different combinations of foods according to the time of the day consumed – a so-called ‘meal pattern’ (17, 18, 20). Recent studies suggest that certain meal patterns – such as skipping meals, longer overnight fasts, and fewer daily eating occasions – may negatively affect health outcomes in older adults. Specifically, skipping breakfast and fasting for over 11 h overnight have been associated with adverse effects on longevity (21, 22).

While research within this area has attracted increased attention (17, 23), the predictive value of meal patterns in relation to all-cause mortality has been explored less often in population-based samples of older adults, leaving a limited understanding of how meal patterns influence mortality risk (23, 24).

Therefore, the aim of this prospective study is to investigate the association between meal pattern variables and 15-year all-cause mortality while also considering relevant covariates in a cohort of 70-year-olds from the Gothenburg H70 birth cohort studies.

## Method

### Study population

Data are derived from the Gothenburg H70 Birth Cohort Studies (H70 studies) in cooperation with the Population Study of Women (PPSW) in Gothenburg (9, 25–28). The H70 studies are multidisciplinary population studies examining birth cohorts of older populations in Gothenburg, Sweden. Participants are systematically recruited using a systematic sampling method, where samples are selected from the Swedish Population Register based on birth dates to yield a representative sample of community-dwelling 70-year-olds. These studies include comprehensive health examinations, focusing on various age-related risk and protective factors, extensively described previously (25–27, 29).

**Fig. 1:**
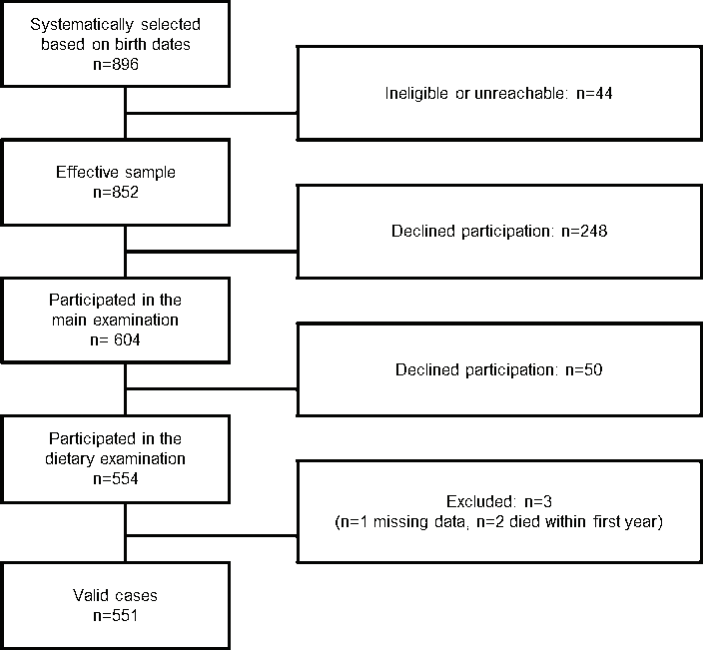
Sample flowchart.

The present study cohort includes individuals born in 1930 who were examined between 2000 and 2002. Initially, 896 individuals were systematically selected and invited to participate. After excluding those who were ineligible (e.g. deceased or emigrated) or unreachable, the effective sample consisted of 852 eligible subjects. Of these, 604 (71%) took part in the main health examinations, which included somatic, neuropsychological, psychiatric, anthropometric, physiological, and genetic tests (Fig. 1). Of the participants in the main examinations, 554 (233 men, 321 women) also completed a diet history (DH) interview. However, 50 participants did not complete the dietary assessment due to various reasons, including impaired cognitive function, difficulties with the Swedish language, early departure during the study day, or the unavailability of a dietician (11). Three individuals were excluded from the final analysis: one due to missing/incomplete dietary data and two due to death within the first year. This resulted in a final sample size of *n* = 551, consisting of 230 men and 321 women. Among the women, 255 were originally recruited to the PPSW (12) and had been followed longitudinally since 1968. The remaining women were recruited in 2000 at age 70 by the H70 study.

Divergence between participants and non-participants has previously been investigated by re-contacting subjects who did not attend the 2000 health examinations (30). The results showed no statistical divergences between participants and non-participants in terms of self-rated health, history of myocardial infarction, diabetes, or smoking status. However, differences were observed in marital status, with a higher proportion of married men among participants (75%) compared to non-responders (56%, *P* < 0.0001), while no substantial differences were noted among women. The most common reasons for non-participation were undefined refusal and illness or ongoing medical care (30).

### Mortality status and follow-up

Mortality status was checked using the Swedish population register on 21 May 2016. The median follow-up time was 15 years (q1–q3 = 12.0–15.2 years). The birth cohort and characteristics of participants and non-participants have been described in detail previously (9, 11, 31). All subjects provided written informed consent, and the Regional Ethical Review Board in Gothenburg approved the studies with reference numbers S227-00 and S377-99. The study was conducted in accordance with the principles of the Declaration of Helsinki.

### Dietary examinations

Dietary intake was assessed using the DH method, which is a semi-structured interview including questions about the usual frequencies and portion sizes of food intake over the preceding 3 months (25, 26). The interviews lasted for about an hour, during which participants were asked to report what they usually eat and drink for each meal and, as far as possible, to specify the food items and amounts consumed. The protocol involved open-ended questions with the aim to illustrate participants’ total intake of food and drinks in an ordinary day. The interviews were conducted face-to-face by trained dieticians. The DH has undergone validation and demonstrated comparable energy intakes (EI) to those predicted by the heart rate method, with some underreporting (12%) observed among overweight participants using double-labelled water (10, 28). Detailed descriptions of the dietary examination procedures have been published previously (12).

To calculate nutrient intake, the Swedish food composition database ‘PC-kost’ (maintained by the Swedish National Food Administration) was utilized. The energy and nutrient variables examined in this study included total daily EI in kcal/day, percentage of energy (E%) from macronutrients and alcohol, and protein intake in grams per day.

### Meal definition and classification

Meal pattern classifications and coding were applied to data derived from the DH interviews. A modified version of Berteus Forslund et al.’s (32) meal classification scheme was used to categorize food intake occasions (IO) into four meal types: light meal/breakfast, main meal, snack meal, and drink-only. The term ‘IO’ is used to describe all types of consumption, including occasions when only beverages are consumed, and ‘eating occasions’ refer solely to instances where solid food is consumed (Table 1). As part of the modifications, for example, ‘breakfast’ was defined as the first eating occasion of the day and consistently coded as ‘light meal/breakfast’, as opposed to the original method wherein the meals were defined by their composition regardless of time of the day.

**Table 1:** Meal pattern chart developed by Bertéus Forslund et al. (32), with descriptions of the meal types (i.e. main meal, light meal/breakfast, snack meal, and drink-only), their composition, and additional grouping terms (i.e. intake or eating occasion). Compared to the original chart, one additional grouping term has been excluded here (i.e. principal meals [main meal + light meal/breakfast])

Type of meal			
Main meal *(e.g. cooked dish, soup with bread, salad with bread, or pizza)*	Light meal/Breakfast *(e.g. porridge, cereal, sandwiches, soup, salad, or omelette)*	Snack meal *(e.g. sandwich, biscuit, bun, cake, fruit, sweet, or ice cream, all with or without a drink)*	Drink only *(e.g. coffee, tea, soft drink, juice, milk, beer, or wine)*

← Intake occasions ➝			
← Eating occasions ➝			

Additional coding instructions were implemented, such as classifying meals involving fewer than two sandwiches as ‘snack meals’ and those involving two or more sandwiches as ‘light meals’. Drinks other than water were coded as individual IO, and overnight fast was defined as the time between the participant’s last eating occasion of the evening and their first eating occasion the next morning, excluding drink-only occasions.

When meals were recorded in a time interval (e.g. 10:00–12:00), the mean time was calculated (e.g. 11:00). Common phrases such as ‘sometimes a bun’ were recorded as 0.5 snack meals. When the frequency of a meal was specified as less than once per day (e.g. 2 times per week), the average intake was calculated per day (0.3 times/day) in order to maximize the coding agreement to allow for future comparison with other cohorts. A logbook was created to gather any uncertain meal descriptions from the DH data, which were audited for agreement.

Several continuous meal pattern variables were categorized to simplify the data representation. Each variable was grouped into specific intervals using cut-off points to define interval groups. For ‘total number of IO’, it was divided into four interval groups: low, medium, medium high, and high frequency. The cut-off points were set as follows: low frequency (≤3), medium frequency (>3–4), medium high frequency (>4–5), and high frequency (>5). Similarly, ‘snack meal’ was binned into four interval groups: low, medium, medium high, and high frequency. The cut-off points for this variable were low frequency (≤1), medium frequency (>1–2), medium high frequency (>2–3), and high frequency (>3). The cut-off points were selected based on variable distribution and the goal of creating balanced groups. For drinks, the cut-off points were <1 and ≥1, while for the main meal variable, the categories were <1, 1, and >1. Regarding overnight fast duration, the first grouping category followed the recommended duration of ≤11 h (33). This group included 25% of the subjects and was further divided into exact quarter groups using the Statistical Package for the Social Sciences (SPSS) binning function. The resulting interval groups were ≤11h referred to as ‘recommended duration’, >11–12.3 h as ‘medium-high duration’, >12.3–13.3 h as ‘high duration’, and >13.3 h as ‘very high duration’.

### Background variables

Participants provided information regarding health variables during a general health examination, (31) including measured body mass index (BMI), self-assessed health situation, nutritional risk indicators, and number of medications. Additionally, socioeconomic variables such as educational level, marital status, and self-assessed economic situation, as well as lifestyle characteristics like cigarette smoking and physical activity level (PAL), were reported at baseline.

BMI (based on measured height and weight values, kg/m^2^) was categorized into three groups: underweight <22, normal weight 22–27, and overweight/obesity >27 according to age-related values presented in the European Society for Clinical Nutrition and Metabolism (ESPEN) guidelines (34). Marital status was dichotomised to married and not married (unmarried/divorced/widowed). Self-rated health was assessed using the general question, ‘How would you rate your health?’ with response options ranging from very good to very poor. Smoking habits were categorized as never smoked, former smoker, and current smoker. ‘Leisure-time physical activity’ and ‘activity level during labour/household work’ were classified on four levels, respectively. The two physical activity variables were, in a second step, quantified into an estimated PAL-value (PAL-value) in accordance with the PAL-estimate template developed and described by Johansson and Westerterp (35). Education was dichotomised into ≤lower than mandatory school and higher than mandatory school.

Risk indicators for undernutrition were defined based on the three assessment points outlined by the National Board of Health and Welfare for assessing nutritional risk, which balances three assessment items: 1) recent weight loss, 2) eating difficulties, and 3) underweight status (36). In this paper, these three assessment points were captured through questions about 1) recent weight/appetite changes, 2) eating/chewing difficulties, and 3) BMI <22. Individuals meeting any of the three criteria were classified as ‘at risk of undernutrition’.

### Statistics

Descriptive statistics are presented as means ± standard deviations (SD) for continuous variables and as counts and percentages for categorical variables. Baseline differences by mortality status were evaluated using independent *t*-tests for normally distributed continuous variables and Mann–Whitney *U* tests for non-normally distributed variables. Categorical variables were analysed using chi-square and Fisher’s exact tests as appropriate.

To examine the association between meal pattern variables and 15-year mortality, Cox proportional hazards regression was used as the primary analytical method. Initially, crude models were applied, followed by two progressively adjusted models (Model 1 and Model 2). Model 1 included adjustments for smoking habits, gender, and self-assessed health status, while Model 2 further incorporated education, EI, economic situation, and marital status. Each categorical variable was compared against a reference group, designated as ‘medium frequency’ for meal pattern variables and ‘recommended duration’ for overnight fasts. Results are expressed as hazard ratios (HR) with corresponding 95% confidence intervals (CI).

As a supplementary analysis, logistic regression models were also conducted, using the same covariates as the Cox regression models, to provide additional insights. These results are discussed briefly in the text but are primarily presented in the supplementary material for reference.

All statistical analyses were conducted using SPSS version 25, with a significance level set at *P* ≤ 0.05.

## Results

Out of the total 551 participants included, 59% were alive at 15-year follow-up and 41% were deceased. For meal pattern frequencies, the total average number of IO was 4.9 per day, of which 1.0 was main meal, 1.9 were snack meals, 1.7 were light meals/breakfast, 0.25 were drink-only occasions, and there were 12 h of overnight fasting.

### Baseline characteristics at 15-year follow-up, stratified by mortality status

Baseline demographic and nutritional characteristics, stratified by mortality status at 15-year follow-up, are presented in Table 2. At baseline, significant differences were observed between those who were deceased at follow-up and those who were living across several characteristics (Table 2). Among those who were deceased at follow-up, a higher proportion were male, current or past smokers, had an education level below mandatory schooling, reported poor self-rated health, and indicated a poor economic situation. Additionally, a greater proportion of those deceased at follow-up had at least one undernutrition risk indicator. This group also showed a significantly lower estimated PAL and used a higher number of medications.

**Table 2:** Background characteristics at baseline (70 years) grouped by 15-year mortality status

	15-year mortality status				
	Living		Deceased		
	*n* Mean	% (SD)	*n* Mean	% (SD)	*P* ^1^
Gender					<0.001*
Female	213_a_	65%	108_b_	48%	
Male	113_a_	35%	117_b_	52%	
Body mass index	26.87_a_	(4.06)	27.22_a_	(4.54)	0.349
Body mass index categories					0.286
Underweight	24_a_	7%	19_a_	9%	
Normal weight	167_a_	51%	98_a_	44%	
Overweight	135_a_	41%	104_a_	47%	
Physical activity level	1.64_a_	(0.08)	1.62_b_	(0.11)	0.003*
Smoking					<0.001*
Never smoked	185_a_	57%	74_b_	33%	
Smoker	35_a_	11%	46_b_	21%	
Former smoker	106_a_	33%	103_b_	46%	
No. of medications	2.93_a_	(2.63)	3.42_b_	(2.96)	0.048*
Health					0.005*
Excellent	88_a_	28%	38_b_	18%	
Good	177_a_	55%	119_a_	56%	
Poor	55_a_	17%	57_b_	27%	
Marital status					0.138
Married	224_a_	69%	139_a_	63%	
Not Married	102_a_	31%	83_a_	37%	
Economic situation					0.063
Excellent	89_a_	28%	52_a_	24%	
Good	207_a_	64%	133_a_	62%	
Poor	25_a_	8%	30_b_	14%	
Education					0.025*
Above mandatory school	140_a_	43%	74_b_	34%	
Below mandatory school	184_a_	57%	146_b_	66%	
Energy intake (kcal)	2155.17_a_	(605.36)	2248.98_a_	(671.20)	0.089
Energy (kcal/kg body weight)	29.40_a_	(8.81)	29.51_a_	(9.74)	0.889
Protein (g/day)	84.54_a_	(22,18)	86.92_a_	(26,21)	0.267
Alcohol (E%)	2.38_a_	(2.78)	3.18_a_	(4.01)	0.082
Undernutrition risk indicators					0.022*
No	245_a_	75%	149_b_	66%	
Yes	81_a_	25%	76_b_	34%	

*Note*: Values in the same row and subtable not sharing the same subscript are significantly different at *P* < 0.05 in the two-sided test of equality for column proportions. Cells with no subscript are not included in the test.

[^1^] Based on independent sample t-tests and chi-square test.

[*] Significant at *P* ≤ 0.05.

No difference in mean BMI or the distribution of BMI categories was shown between the groups. Similarly, no differences were found for total EI (kcal), EI per kilogram of body weight, and protein intake (g/day). However, when stratified by sex (data not shown), a significant difference in average protein intake (g/day) was observed between living and deceased women (M = 78.4, SD = 18.8; M = 73.2, SD = 19.0; *P* < 0.021).

### Meal pattern characteristics at baseline by mortality status

Student’s *t*-test (data shown in the supplementary file) revealed that the daily number of IO had a similar average of approximately 5 in both groups, regardless of mortality status. Similarly, the average number of snack meals per day was about 2 in both groups. The total number of eating occasions and light meals/breakfast was slightly lower among deceased subjects compared to those alive. However, Student’s *t*-test indicated no significant differences in the continuous meal pattern variables, except for drink-only occasions, where the deceased group demonstrated a higher average intake (0.21 vs. 0.31; *P* = 0.04).

Regarding categorical meal pattern intervals (Table 3), a somewhat lower proportion of deceased subjects were categorized in the medium (>3–4) IO interval and a somewhat lower proportion of alive subject was in the ‘≥1’ drink-only interval compared to those deceased at follow up. Chi-square tests showed that the drink categories were the only variable that showed significant relations between deceased and alive *X*^2^(1, *N* = 551) = 4.2, *P* = 0.04.

**Table 3:** Baseline (70 years) categorical meal pattern variables grouped by 15-year mortality status

	15-year mortality status				
	Living		Deceased		
	*n*	%	*n*	%	*P* ^1^
Intake occasions grouped					0.188
Low ≤3	13_a_	4	13_a_	6	
Medium >3–4	100_a_	31	51_b_	23	
Medium high >4–5	121_a_	37	92_a_	41	
High >5	92_a_	28	69_a_	31	
Main meals grouped					0.773
<1	298_a_	91	203_a_	91	
=1	9_a_	3	5_a_	2	
>1	19_a_	6	16_a_	7	
Snack meals grouped					0.277
Low ≤1	98_a_	30	74_a_	33	
Medium >1–2	128_a_	39	71_a_	32	
Medium high >2–3	75_a_	23	63_a_	28	
High >3	25_a_	8	17_a_	8	
Drink-only grouped					0.041*
<1	272_a_	83	172_b_	76	
≥1	54_a_	17	53_b_	24	
Overnight fast grouped					0.943
Medium ≤11.00	99_a_	30	64_a_	28	
Medium high >11–12.00	71_a_	22	51_a_	23	
High >12.15–13.00	80_a_	25	59_a_	26	
Very high >13.00	76_a_	23	51_a_	23	

*Note:* Values in the same row and subtable not sharing the same subscript are significantly different at *P* < 0.05 in the two-sided test of equality for column proportions. Cells with no subscript are not included in the test.

[^1^] based on chi-square test.

[^*^] significant at *P* ≤ 0.05, not adjusted for covariates.

### Meal pattern analysis and mortality risk: Cox regression

Cox proportional hazards regression analyses revealed that higher intake frequencies were associated with an increased risk of mortality compared to the reference category. In the fully adjusted model, individuals with 4–5 and more than 5 IO per day had a significantly higher hazard of 15-year mortality (HR = 1.51, *P* = 0.02 and HR = 1.56, *P* = 0.02, respectively) compared to those in the reference group with 3–4 IO per day (Table 4).

**Table 4:** Cox regression models using meal pattern variables as predictor for 15-year mortality

	Crude				Model 1^a^				Model 2^b^			
	B	HR	(95% CI)	*P*	B	HR	(95% CI)	*P*	B	HR	(95% CI)	*P*
Intake occasion groups												
References: 3–4												
<3 Low	0.61	1.85	(0.98–3.47)	0.06	0.60	1.82	(0.97–3.42)	0.06	0.52	1.68	(0.87–3.24)	0.12
>4–5 Medium high	0.37	1.45	(1.03–2.04)	0.04*	0.40	1.49	(1.04–2.14)	0.03*	0.41	1.51	(1.05–2.16)	0.03*
>5 High	0.30	1.35	(0.93–1.94)	0.11	0.41	1.51	(1.04–2.20)	0.03*	0.44	1.56	(1.07–2.27)	0.02*
Snack meal groups												
References: 1–2												
<1 Low	0.28	1.32	(0.95–1.84)	0.10	0.40	1.49	(1.06–2.10)	0.02*	0.40	1.49	(1.06–2.11)	0.02*
>2–3 Medium high	0.31	1.37	(0.97–1.93)	0.07	0.42	1.51	(1.06–2.16)	0.02*	0.41	1.51	(1.05–2.17)	0.03*
>3 High	0.16	1.17	(0.69–2.00)	0.56	0.43	1.54	(0.89–2.68)	0.13	0.47	1.59	(0.91–2.78)	0.10
≥1 Drink meal	0.35	1.41	(1.04–1.93)	0.03*	0.32	1.38	(1.00–1.90)	0.05*	0.30	1.35	(0.98–1.86)	0.68

CI, confidence interval.

a Model 1, the included variables are health situation, smoking habits, and gender.

b Model 2, the variables of Model 1 as well as education, EI, economic situation, and marital status are included as covariates.

[^*^] Significant at *P* ≤ 0.05.

Regarding snack meals, the Cox regression analyses showed significant associations for both the ≤1 and 2–3 snack meals per day groups, with HR of 1.49 (*P* = 0.022) and 1.51 (*P* = 0.025), respectively, compared to the reference group of more than 1–2 snack meals per day.

In contrast, no significant associations were observed between drink-only occasions and 15-year mortality in the fully adjusted Cox model.

**Fig. 2:**
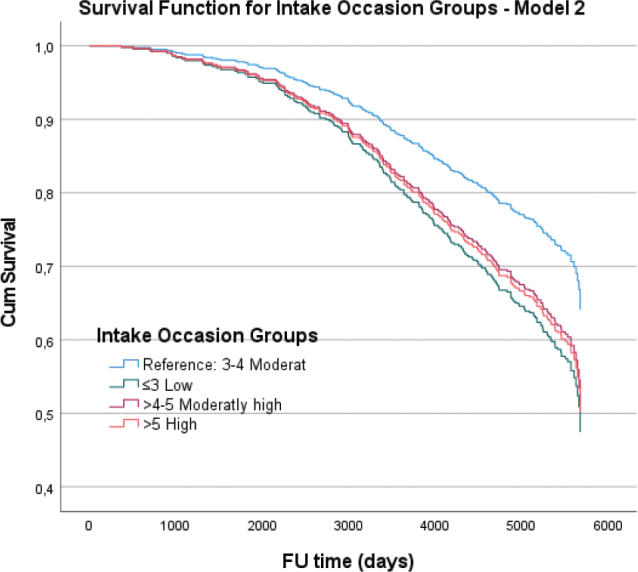
The cumulative survival functions for different IO Groups in the fully adjusted model, including covariates such as health status, smoking habits, gender, total EI, marital status, and education level (Model 2). Each plot compares the survival distributions against a reference group with medium IO frequency (3–4 times per day), illustrating the impact of these variables on survival over a follow-up period of up to 6,000 days.

**Fig. 3:**
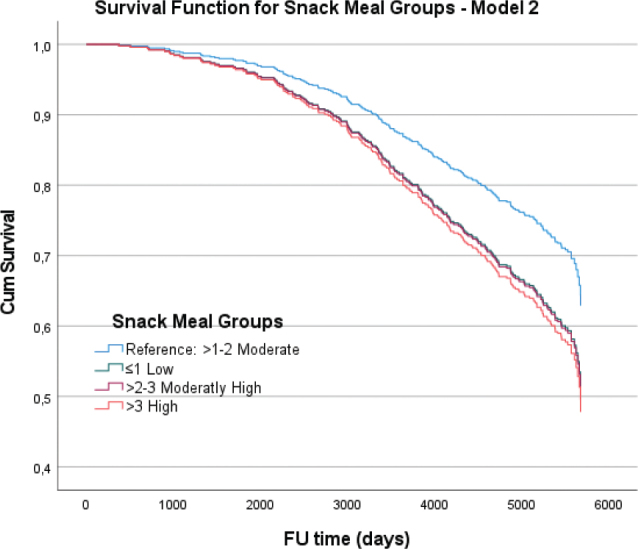
The cumulative survival functions for different ‘Snack Meal Groups’ in the fully adjusted model, including covariates such as health status, smoking habits, gender, total EI, marital status, and education level (Model 2). Each plot compares the survival distributions against a reference group with medium snack meal frequency (>1–2 times per day), illustrating the impact of these variables on survival over a follow-up period of up to 6,000 days.

Overall, these analyses indicate a higher HR associated with more frequent intake intervals, particularly for those beyond 4–5 occasions per day, even after adjusting for EI and other covariates. Figures 2 and 3 illustrate the cumulative survival functions for different IO groups and snack meal groups, respectively, across the Cox regression models, highlighting the impact of meal frequency on survival over a follow-up period of up to 6,000 days.

## Discussion

We examined the meal pattern in relation to 15-year all-cause mortality in a population-based sample of community-dwelling 70-year-olds, first examined 2000–2002 and followed up in 2015. The study is part of the Gothenburg H70 birth cohort studies. We found significant relationships between meal pattern variables and increased risk of mortality. At follow-up, 59% of the participants remained alive. In line with official health statistics of Sweden (37), deceased subjects were to a greater extent male, current/past smokers, had lower education levels, reported worse self-assessed health, and indicated a poorer economic situation compared to those who remained alive. Additionally, there was a higher proportion of subjects with risk indicators for undernutrition among those deceased at follow-up compared to those alive (Table 2). No significant differences in meal pattern variables were observed in relation to mortality status based on bivariate analyses, except for drink-only intakes, which were more frequent among those deceased at follow-up (Table 3). However, Cox regression analyses using categorical meal pattern variables revealed significant associations between higher total IO and increased mortality risk, as well as between snack meal frequencies and mortality (Table 4). These associations remained significant even after adjusting for EI and other covariates.

The World Health Organization (WHO) recommends 5–6 small non-fatty meals for older adults (2). A meal pattern associated with greater food variety and lower body fat, blood glucose, and lipid levels, especially if larger meals are eaten early in the day. The Swedish Food Agency recommends three main meals and at least three snack meals per day and an overnight fast not exceeding 11 h within the care of frail older adults to counteract undernutrition (36). Several smaller meals distributed over more hours make it easier to cover energy and nutrient needs, particularly for those with poor appetites (38). These recommendations target older adults, but from different perspectives. The aim of the WHO recommendations is metabolic control, while the Swedish recommendations focus on the risk of undernutrition. The new Nordic Nutrition Recommendations (NNR) 2023 (39) state that there is not enough evidence to set dietary guidelines on meal patterns and underscore the variability of meal patterns within the context of an energy-balanced and nutritionally adequate diet (39). This perspective aligns with our interpretation of the findings, suggesting that while meal patterns may influence mortality risk, they should not be viewed in isolation from the nutritional quality of the diet.

As stated in the section titled ‘Introduction’, older adults as a group are characterized by large heterogeneity in terms of health and vigour. Therefore, recommendations to decrease the risk for metabolic conditions such as cardiovascular disease are relevant for some part of the ageing population at the same time as recommendations to decrease the risk of undernutrition are relevant for another part. The current sample was derived from the general population of 70-year-olds in Gothenburg and consisted of older adult participants generally in quite good health and not comparable to frail seniors, to whom the dietary advice above from the Swedish authorities applies. However, despite the generally good health status, risk indicators for undernutrition were present in both groups but significantly more prevalent among the deceased compared to individuals alive at follow-up at age 85.

### Integrating findings with existing literature on meal patterns and health

Putting our results in the context of the present science with regard to meal frequency, a narrative literature review in various populations showed that the most common temporal meal patterns, irrespective of dietary intake method, were a ‘3 meals/day pattern’, a ‘skipping breakfast pattern’, and a ‘nibbling’ pattern consisting of smaller but more frequent meals (40). Initial research largely suggested that higher meal frequency might offer protective effects against metabolic risk factors such as weight gain and diabetes (41–47). Conversely, more recent longitudinal studies indicate that frequent meals and snacks may increase the risk of adverse health outcomes, including coronary heart disease (CHD), weight gain, and type 2 diabetes (48–51). For instance, Cahill et al. (50) conducted a prospective cohort study examining meal frequency and CHD risk in older men. Although their results were not statistically significant, they identified a trend: men consuming 1–2 meals daily had a relative risk (RR) of CHD of 1.10, those eating 4–5 meals had an RR of 1.05, and men eating six or more meals daily had an RR of 1.26, compared to a baseline of 3 meals per day, after adjusting for total EI, diet composition, and other risk factors.

Our findings contribute to this complex picture. In the crude analysis, we found no significant association between total daily IO and 15-year mortality. However, when controlling for factors such as subjective health, smoking habits, and gender, our adjusted models revealed that individuals consuming more than five meals daily faced an increased hazard of mortality over the 15-year period. This consistent trend across models suggests a potential risk associated with higher meal frequencies, aligning with Cahill et al.’s observation of elevated CHD risk in men eating six or more meals per day (50).

Contrastingly, our analysis also found that certain intake frequencies – specifically, the consumption of 2–3 snack meals per day – were consistently associated with a higher hazard of mortality, regardless of the model adjustments. While our study focused primarily on meal frequency and type, these findings highlight the need for future research to incorporate meal composition alongside meal patterns to gain a more comprehensive understanding of their relationship with health outcomes.

Moreover, the interplay between meal frequency and nutritional quality cannot be overlooked. For example, the relations between meal frequencies and weight gain have shown a positive association with higher sugar intake (23, 48, 49), and it is suggested that increased food stimuli by several meals per day may increase the desire to overeat. According to Paoli et al. (23), the contrasting results on meal frequency and health can be explained by the fact that a reduced number of meals often reflects a disadvantageous distribution, such as skipping breakfast, having a light lunch, and a calorie-dense dinner, which can lead to poor metabolic control (52). This highlights the importance of distinguishing between individuals who follow a healthy eating pattern despite fewer meals and those whose reduced meal frequency is due to disadvantageous habits.

Our study aligns with current research, which presents conflicting results on the relationship between meal patterns and health outcomes. These discrepancies likely arise from variations in populations and differing methods of classifying meal patterns. While meal frequency is influential, it is not the sole determinant of health impacts. The nutritional content of meals, particularly the quality and balance of macronutrients, plays a critical role in shaping long-term health trajectories. For example, replacing protein with simple carbohydrates like sugars alters metabolic response, affecting both short- and long-term health (48, 53). Shang (54) demonstrated that higher protein and fat intake at breakfast, combined with lower carbohydrate intake, was associated with a lower rate of cognitive decline over 9 years in older adults. Their study also found that high-frequency diets substituting carbohydrates with protein may help delay or prevent cognitive decline, likely due to better glycaemic control and reduced postprandial insulin responses (48). Additionally, the quality of fat is important for both dementia and cardiovascular disease risks (55). Thus, our research emphasizes the need for a nuanced approach in public health strategies that extends beyond meal frequency to include a holistic consideration of dietary composition.

## Strength and limitations

Strengths in the present study include its population-based study design, the use of an age-standardized sample, and a prospective approach spanning 15 years. These features are particularly valuable in a research field where data on the long-term health effect of meal frequency in older adults remains limited. The method for meal type classifications used was a modified version of Berteus Forslund et al. (32), using a predetermined meal definition which increases the standardization of the coding. Inter-observer variations were minimized as one person coded all data. However, meal patterns could be classified by different methods, meaning that comparison with other studies may involve uncertainty (17).

A further strength is adjustment for relevant covariates. In the first model, gender, self-assessed health, and smoking were included, all of which were significant across the analysed meal pattern variables. The second model additionally included total EI, education, economic situation, and marital status (Table 4). While not all of the variables in the final model, they are well established as important covariates in previous studies examining dietary habits and health.

However, predicting mortality by analysing meal frequency does not stand alone. Additional information on food and nutrient composition is important to understand the complete health effect of meal patterns. Therefore, it is a limitation that we only could report protein intake and alcohol expressed as energy %.

Another limitation regarding generalizability is the participation rate at baseline, which was 61%. Population-based studies often face challenges related to positive self-selection bias, as individuals who choose to participate may be healthier or more motivated than non-participants. This may result in an under-representation of individuals with poorer health or lower socioeconomic status. A positive association between dietary quality and socioeconomic status at age 70 has previously been demonstrated in this cohort (56), highlighting the potential influence of socioeconomic factors on both meal patterns and mortality outcomes. However, baseline differences between those deceased and alive at follow-up were consistent with official health statistics of Sweden (14, 57), suggesting that the cohort reflects broader population trends. It is also worth noting that gender distribution was skewed, with women being over-represented – an expected outcome given their longer life expectancy compared to men (57). While this reflects demographic realities, it may limit the generalizability of findings to men. It is also important to consider that individuals with impaired cognitive function or language difficulties were more likely to be excluded from dietary assessments, which could introduce selection bias related to meal patterns and mortality risk.

## Future perspectives

Based on the present results and existing evidence for the association between meal frequency and health in older adults, future studies might include nutritional and food composition of different types of meals, which might add deeper understanding of the effect of meal pattern on health (58).

## Conclusions

The 15-year follow-up in this study offers a comprehensive and long-term perspective on the relationship between meal patterns and longevity among older adults. Our results indicate that frequent daily consumption of snacks or total intake episodes is associated with increased risk of mortality between 70 and 85 years, independently of the amount of energy consumed. Specifically, individuals with high total intake frequencies (more than five IO per day) and those with snack frequencies of two to three snacks per day exhibited a significantly elevated risk of 15-year mortality.

Given the implications of these findings, further research is warranted to explore the underlying mechanisms linking meal frequency and mortality. Detailed investigations into the nutritional composition of different meal patterns, as well as their metabolic and physiological impacts, could provide deeper insights into how meal frequency affects health. Therefore, future studies should consider the potential influence of meal timing, quality of food consumed, and individual metabolic responses.
